# Warning to Chicago and Vicinity (and to Texas)

**Published:** 1899-02

**Authors:** Arthur R. Reynolds

**Affiliations:** Commissioner of Health; Department of Health, City of Chicago


### Warning to Chicago and Vicinity (and to Texas).

DEPARTMENT OF HEALTH, CITY OF CHICAGO, Jan. 29, 1899.

*To the Public:*

Smallpox is spreading throughout the world to an extent not equaled since 1893. *Public Health Reports,* the official organ of the Marine Hospital Bureau, our national health service, records the recent invasion of nineteen States and sixty-three localities by the pestilence in this country alone, and the newspapers add daily to the number. Some of these localities are within a few hours' distance of Chicago, and it is not only possible that there are concealed or unrecognized cases of the disease in the city at the present time, but it is more than probable that persons from infected localities are daily riding in our street cars, associating with our citizens in public places, and even visiting in our homes.

When the contagion becomes so widespread as it now is, it is obviously impossible to prevent its introduction into a new city like Chicago, with its numerous means of communication with the rest of the country. Sooner or later the disease is sure to find its way into the city by some unsuspected and unpreventable method. Once introduced—especially at this season of the year—its spread among the susceptible is inevitable.

No degree of cleanliness of the individual, or of hygiene in the household, or of municipal sanitation will protect the susceptible against contracting smallpox if exposed to its contagion. The one certain and only safeguard—tested by the experience of the century—is effective vaccination.

As commissioner of health, charged with the protection of health and life, upon which our municipal welfare and prosperity depend, I earnestly urge upon every citizen to see to it that he himself and those dependent upon him are thoroughly and promptly protected against this loathsome pestilence. Parents and guardians cannot escape the charge of blood-guiltiness if, failing to secure this protection for their charges, death follows an attack of smallpox—a disease of all diseases the most positively preventable.

Employers and others having charge of wage-workers, especially garment-makers and textile-fabric handlers, should give this matter attention, from pecuniary considerations if no other. Similarly as to railway employes, who are particularly exposed, and the employes of manufacturing establishments, department stores, etc. It should be made a condition of further employment that all such persons present satisfactory evidence of protection against smallpox by recent successful vaccination or revaccination.

The remembrance of the late smallpox epidemic, that of 1893-4, should be still fresh in the public mind; but few, probably, realize its cost to the community. The 3076 cases and 1033 deaths of that epidemic represent, according to the eminent authority, Dr. Benjamin Lee, a money loss of $2,581,000, to which must be added the enormous losses from damage to business, suspension of industries, interruption of travel and traffic, and injury to commercial reputation.

It only remains to add that the glycerinated vaccine of the health department is now at the highest standard of purity and efficiency; that its proper use causes the minimum of discomfort and insures absolute freedom from any danger of infection of other diseases as well as from the painful "sore arms," unsightly scars, and other drawbacks which attended the former use of vaccine "points."

While the department prefers that the vaccination should be done by private physicians, it is ready and anxious to vaccinate without charge all who are unable to pay for the operation.

Arthur R. Reynolds, M. D.,
Commissioner of Health.

---

**The** *Kansas Medical Journal*, which has been published for the last ten years in Topeka, Kansas, has been discontinued, and its former editor, Dr. W. E. McVey, will have editorial control of the *Medical Monograph*, a 150-page monthly, which will be published in the place of the *Kansas Medical Journal*.

